# Prevalence of Korean cats with natural feline coronavirus infections

**DOI:** 10.1186/1743-422X-8-455

**Published:** 2011-09-28

**Authors:** Dong-Jun An, Hye-Young Jeoung, WooSeog Jeong, Jee-Yong Park, Myoung-Heon Lee, Bong-Kyun Park

**Affiliations:** 1 National Veterinary Research and Quarantine Service, Anyang, Kyunggi-do, 430-824. Korea; 2 Department of Veterinary Medicine Virology Lab, College of Veterinary Medicine, Seoul National University, Seoul, 151-742, Korea

**Keywords:** FCoV I, FCoV II, Seroprevalence

## Abstract

**Background:**

Feline coronavirus is comprised of two pathogenic biotypes consisting of feline infectious peritonitis virus (FIPV) and feline enteric coronavirus (FECV), which are both divided into two serotypes. To examine the prevalence of Korean cats infected with feline coronavirus (FCoV) type I and II, fecal samples were obtained from 212 cats (107 pet and 105 feral) in 2009.

**Results:**

Fourteen cats were FCoV-positive, including infections with type I FCoV (n = 8), type II FCoV (n = 4), and types I and II co-infection (n = 2). Low seroprevalences (13.7%, 29/212) of FCoV were identified in chronically ill cats (19.3%, 16/83) and healthy cats (10.1%, 13/129).

**Conclusions:**

Although the prevalence of FCoV infection was not high in comparison to other countries, there was a higher prevalence of type I FCoV in Korean felines. The prevalence of FCoV antigen and antibody in Korean cats are expected to gradually increase due to the rising numbers of stray and companion cats.

## Background

Feline coronavirus (FCoV) is an enveloped, positive-sense, single-stranded RNA virus of the family Coronavirideae within the order Nidovirales. FCoVs are comprised of two pathogenic biotypes [1] consisting of feline infectious peritonitis virus (FIPV) and feline enteric coronavirus (FECV), which are both divided into two serotypes (Type I and II). The serotypes differ in their growth characteristics in cell culture and antigenicity, relative to canine coronaviruses (CCoVs) [2].

The prevalence of type I and II FCoV have been surveyed in many countries, including Japan [3], United States [4], United Kingdom [5], Austria [6], Switzerland [7], and Taiwan [8]. The majority of field isolates in these countries are of type I, regardless of the assay method [4].

FCoVs are associated with mild or subclinical enteric infections [9]. However, in a small proportion of cats, FCoV infection leads to the development of a lethal, immune-mediated condition known as feline infectious peritonitis (FIP) [10,11]. FIP is a complex immune disease involving virus or viral antigen, antiviral antibodies, and complement. Cats that do not develop anti-FCoV antibodies do not develop FIP.

Serological surveys of FCoV infection have involved detection of antibody by indirect fluorescent antibody assay (IFA) or enzyme-linked immunosorbent assay (ELISA) [12-15]. A plaque-reduction neutralization test (PRNT) was developed to serologically distinguish FCoV type I and II infections in cats [16].

In a previous study, the number of cats raised in Seoul, the capital city of South Korea, was reported to be around 30,000 in 2004 [17]. The most common breed in Korea is the Korean short hair cat, but this has been changing the recent years due to the increasing number of cats that are being raised as companion cats. Previously, there have been case reports of FIP in a Persian chinchilla (2 years age, female) and a Korean short hair cat (3 months age, male) [18], but no nationwide survey for FCoV has been carried out in Korea.

Therefore, the objectives of the current study were to identify the seroprevalence of FCoV and to classify the FCoV serotypes in Korean cats in comparison with prevalence in other countries.

## Methods

### Cat specimens

Feline serum and fecal swab samples were collected from 212 cats consisting of 107 samples from six local animal hospitals (four in Seoul and two in Kyunggi) and 105 samples from two animal shelters (Incheon and Daejeon) in 2009. Of the 212 samples, 129 were from clinically healthy cats and 83 were from cats displaying symptoms of illness that included inappetence, anorexia, weight loss, lethargy, icterus, fever, diarrhea, and thoracic effusion.

### RNA extraction and reverse transcription-polymerase chain reaction (RT-PCR)

Viral RNA was extracted from fecal samples using TRIzol LS^b ^(Invitrogen, Valencia, CA) according to the manufacturer's instructions. Two RT-PCR methods were used: one enabled the highly sensitive detection in fecal samples and the other distinguished type I from type II. The former method was based on nested PCR using primers for the amplification of the highly conserved 3'-untranslated region (3'-UTR) of the FCoV genome [19]. The latter method [8], which was based on the spike gene region of the FCoV genome, is a multiplex-nested PCR using 2 μL of the first PCR product as the template and nested primers [5] for discrimination of Type I and II FCoVs. In addition, FCoV positive samples were further tested for feline panleukopenia virus (FPLV) using a specific FPLB primer set and conditions previously described [20] to identify possible relationships between the two viruses.

### IFA detection of anti-FCoV antibody

Anti-FCoV antibody titers in feline serum samples were determined by IFA using porcine kidney-15 (PK-15) cells and porcine transmissible gastroenteritis virus (TGEV) [19]. In brief, PK-15 cells were inoculated with TGEV at a multiplicity of infection of 10 plaque forming units (PFU)/cell and incubated for 8 h at 37°C. PK-15 cells were trypsinized, mixed approximately half-and-half with uninfected cells, and seeded into wells of eight-well glass plates (Nutacon, Amsterdam, The Netherlands). Cells were incubated for 60 min at 37°C with serial two-fold dilutions of serum samples in phosphate buffered saline (PBS), washed three times for 5 minutes, and incubated for 1 hour with fluorescein isothiocyanate-conjugated goat anti-feline immunoglobulin G (KPL, Gaithersburg, MD).

### Phylogenetic analysis

Korean FCoV sequences from this study and 30 sequences from global FCoV isolates obtained from GenBank were aligned using the Clustal X 1.83 sequence alignment program [21]. Nucleotide and amino acid sequence identities among the Korean FCoV isolates were calculated using BIOEDIT 7.053 [22]. The phylogenetic tree was created by a neighbor-joining method using MEGA 4.0 software [23].

## Results

The total number of positive fecal samples for FCoV 14 cats, which were mostly Korean short hair breeds over one year of age. Of these FCoV positive cats, three were identified to be co-infected with FPLV (Table 1).

**Table 1:** Summary for 14 cats with Feline Coronavirus type I, and II, or co-infection.

Cat No	Breed	Gender	Age (month)	Region	Collection Site	Collection Month	FCoVs type	Strain	Accession No	Antibody titer (IFA)	FPLV
420	Domestic short hair	M	12	Kyonggi	Hospital	April	I	08K-420	JN654401	80	-
478	Domestic short hair	M	9	Seoul	Hospital	April	I	08K-478	JN654402	640	-
559	Domestic short hair	M	30	Kyonggi	Hospital	April	I & II	08K-559/I 08K-559/II	JN654403 JN654411	160	-
609	Domestic short hair	F	14	Seoul	Hospital	May	II	08K-609	JN654412	20	-
656	Scottish Fold	F	12	Daejeon	Shelter	May	II	08K-656	JN654413	640	-
958	Domestic short hair	M	12	Incheon	Shelter	June	I	08K-958	JN654404	40	-
1011	Scottish Fold	M	42	Seoul	Hospital	June	I	08K-1011	JN654405	20	+
1154	Domestic short hair	F	11	Daejeon	Shelter	July	II	08K-1154	JN654414	80	-
1177	Perisan	M	14	Kyonggi	Hospital	July	I	08K-1177	JN654406	320	-
1429	Scottish Fold	F	15	Seoul	Hospital	Aug	I & II	08K-1429/I 08K-1429/II	JN654407 JN654415	80	-
1549	Domestic short hair	M	10	Incheon	Shelter	Sep	I	08K-1549	JN654408	640	-
1552	Domestic short hair	M	12	Seoul	Hospital	Sep	I	08K-1552	JN654409	20	+
1553	Domestic short hair	F	24	Seoul	Hospital	Sep	II	08K-1553	JN654416	160	-
1991	Domestic short hair	M	24	Incheon	Shelter	Dec	I	08K-1991	JN654410	80	+

Among the 212 feline fecal swab samples, eight were positive for FCoV type I, four were positive for type II, and two were positive for types I and II (Table 2). Eleven of the positive samples were from cats displaying symptoms of infection. Three of the positive samples were from asymptomatic cats (Table 2). Among the ill cats, the infections were due to type I (n = 6), type II (n = 3) and both types I and II (n = 2). Of the three positive samples from asymptomatic cats, two were type I and one was type II (Table 2). Of the 107 specimens collected from six local animal hospitals, the nine FCoV-infected cats included type I (n = 5), type II (n = 2), and types I and II (n = 2) (Table 1). The 105 fecal swab samples obtained from two animal shelters included five that were FCoV positive (three type I and two type II; co-infection was not evident).

**Table 2:** Prevalence of feline CoV in Korea by type and clinical status.

Clinical status	Number of case tests			
	
		Typable		Total
		
	Type I	Type II	Type I and II	
Ill	6/83 (7.2%)	3/83 (3.6%)	2/83 (3.6%)	11/83 (13.5%)

Healthy	2/129 (1.6%)	1/129 (0.8%)	0/129 (0%)	3/129 (2.3%)

Total	8/212 (3.8%)	4/212 (1.9%)	2/212 (0.9%)	14/212 (6.6%)

**Figure 1:**
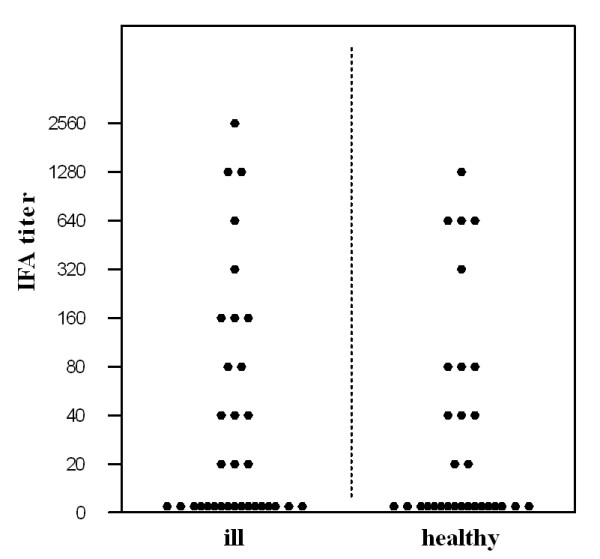
**Distribution of immunoflurorecent assay (IFA) titers to FCoV among cats**. IFA titers are expressed as the reciprocal value of the highest dilution. On the left half are chronically ill cats and on the right half are healthy cats.

Alternatively, canine coronavirus (CCoV) or TGEV can be used as a target antigen because of their close serological identity with FCoV. The prevalence of anti-FCoV antibody in the feline samples was low (29/212; 13.7%) and comprised 20-fold (n = 5), 40-fold (n = 6), 80-fold (n = 5), 160-fold (n = 3), 320-fold (n = 2), 640-fold (n = 4), 1280-fold (n = 3), and 2560-fold (n = 1) (Figure 1). The 29 cats having FCoV antibodies included 16 of the 83 ill cats (19.3%) and 13 of the 129 healthy cats (10.1%), and, according the location of sample collection, represented animal hospital cats (14.0%, 15/107) and shelter cats (13.3%, 14/105).

**Figure 2:**
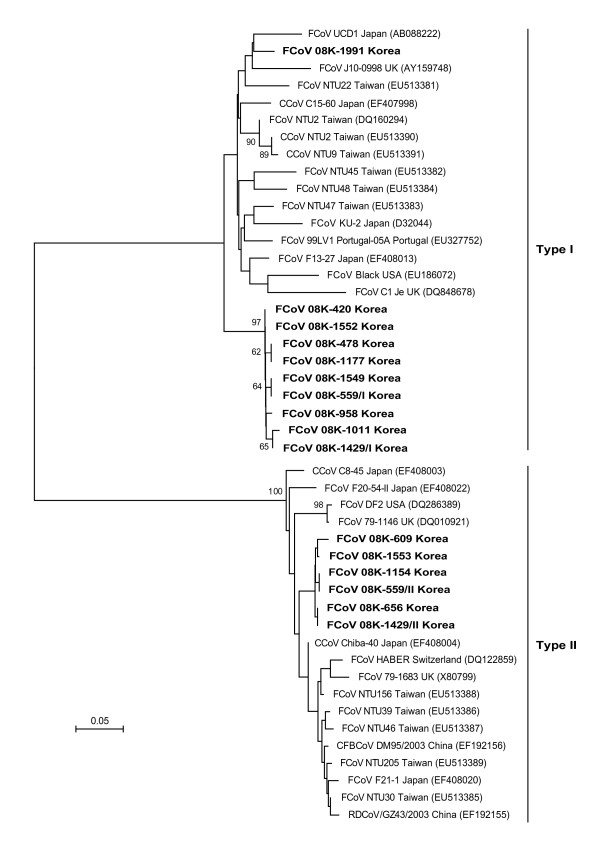
**Phylogenetic relationships between partial spike gene sequences of 46 coronaviruses**. The samples were comprised of 16 FCoVs from Korea, 23 FCoVs from around the world, five CCoVs, one RDCoV, and one CFBCoV. All sequences except the 16 FCoV sequences derived from the current study (in boldface) were obtained from GenBank. An unrooted phylogenetic tree was constructed from aligned sequences by the neighbor-joining (NJ) method using MEGA 4.0 software. Bootstrap percentages are shown above those branches that are supported in at least 60% of the 1,000 replicates. Scale bar indicates nucleotide substitutions per site.

A phylogentic analysis was performed using spike gene partial sequences from 46 CoVs including Korean FCoV from the current study (n = 16), FCoVs obtained globally (n = 23), CCoVs (n = 5), raccoon dog CoV (RDCoV; n = 1), and Chinese fetter badger CoV (CFBCoV; n = 1). The phylogenetic tree data set was resampled 1000 times to generate bootstrap percentage values. The phylogenic tree clearly delineated types I and II FCoVs. The analysis showed that of the ten Korean FCoVs in the type I group, nine type I FCoVs belonged to a different lineage when compared to strains isolated from other parts of the world, and only the Korean FCoV 08K-1991 was grouped with UCD1 (Japan). All six Korean type II FCoVs (08K-609, 08K-656, 08K-1154, 08K-1553, 08K-559/II and 08K-1429/II) was shown to be originated from the same lineage as F20-54-II isolated from Japan (Figure 2).

## Discussion

The prevalence of type I and II FCoVs has been surveyed in many countries [3-8,16]. Similar to these other countries, the majority of FCoV infections in Korea was shown to be of type I (66.7%), with type II constituting approximately 33.3%. The type I virus is more genetically diverse than type II, perhaps reflecting its global distribution and a long period of persistence of the virus in asymptomatic cats [5,24].

Previous studies have reported the proportion of infected cats that are co-infected with type I and II viruses were as high as 15.8% (6/38) in Taiwan [8] and 6.8% (5/74) in Austria [6]. In the current study, there was no incidence of co-infection with type I and II FCoV in healthy cats, whereas the prevalence of co-infection in ill Korean cats was somewhat higher (14.3%, 2/14) than that reported in cats from Austria. Investigation of both of the co-infected Korean cats revealed that they dwelled in a house that also had a dog as a companion animal. This indicates that type II FCoV may have originated from a double recombination between type I FCoV and type II CCoV [25].

Previous research conducted in several countries has revealed that 69-91% of seropositive cats are suspected of having a type I infection [3,4,7]. In the present study, although the evaluation for seroprevalence by the virus neutralization test between types I and II FCoV could be not performed, FCoV seroprevalence was presently somewhat lower than the previously reported prevalences from other countries.

In a study conducted in Japan in the early 1990s, a low prevalence of FCoV antibodies was detected in chronically diseased cats (21.3%) and non-diseased cats (13.4%) by IFA [3]. A more recent study in that country that utilized PRNT reported a substantially greater (three times) seroprevalence [16]. The latter authors suggested that, despite the differing analysis regimens and samples, type II FCoV infection has been decreasing in Japanese domestic cats in recent years. Accordingly, we anticipate that the use of the PRNT array for Korean feline specimens would produce a higher seroprevalence than was evident using IFA.

Phylogenic analysis of the spike gene of FCoV types I and II clearly discriminated between the two types (Figure 2), in agreement with previous results [8]. The 16 Korean FCoVs in our study were classified into two serotypes, type I (n = 10) and II (n = 6) on the neighbor joining tree which was inferred using the Kimura 2 parameter model assuming uniform rates of change among sites. Geographical origin or sub-lineage classification of the FCoVs was not evident due to low nodal support value, but the perfect bootstrap percentage on the node (CCoV C8-45) indicates that our strains may have originated from this type II prototype.

In this study, we examined the prevalence of Korean cats infected with type I and II FCoV and identified the seroprevalence of FCoV in Korean cats. Type I and II FCoV are not highly prevalent in comparison to the prevalence of other countries. However, in recent years Korean feline population, has been rising and it's possible their occurrence may increase in the future.

## Competing interests

The authors declare that they have no competing interests.

## Authors' contributions

DJA: design of the experiments and writing the manuscript, JYP: help in writing the manuscript, WJ and MHL: conducted data analysis, HYJ: conducted PCR for FCoV, BKP: final correction of the manuscript. All authors read and approved the final manuscript.
